# Supplementary material for: Differential Impact of Intermittent vs. Sustained Hypoxia on HIF-1, VEGF and Proliferation of HepG2 Cells
Source: Int J Mol Sci. 2023 Apr 7;24(8):6875. doi: 10.3390/ijms24086875 (PMC10139223; doi:10.3390/ijms24086875)
Supplement: Supplementary file 1 [file ijms-24-06875-s001.zip › ijms-2303705-supplementary.pdf]

Supplementary Materials:

- **Supplementary data**

QPCR Primers

Gene expression was normalized to HPRT1 and RPLP0 rRNA content as internal controls and expressed as fold change compared with control. The primers sequences associated with the 3 genes tested are summarized were as follows:,with the following qPCR program: 1 cycle: 15min, 95°C, 40 cycles: 15sec-94°C, 30sec-60°C, 30sec-72°C, 1 cycle: 1min-95°C, 30sec-60°C, 30sec-95°C.

Gene expression was quantified using the comparative threshold cycle (Ct) method14 with a normalization against HPRT1 (F-TCTTTGCTGACCTGCTGGATTACAT, R-CCAGGGAAAGCAAAGTTTGCATT) and RPLP0 (F-CCCTGCACTCTCGCTTTCTGGA, R- AGGGGCAGCAGCCGCAAATG). HIF-1α (Hif1a F- CGGCGACATGGTTTACATTTCTG, R-CTTTTCTCACTGGGCCATTTCTGT), endothelin-1 (Edn1, F-CGTACCGTATGGACTGGGAG, R- GAGCGCACTGACATCTAACTG), VEGF-A (Vegfa F- CTTCAAGCCATCCTGTGTGC, R-TTGCTCTATCTTTCTTTGGTCTGC).

- **Supplementary Figures**

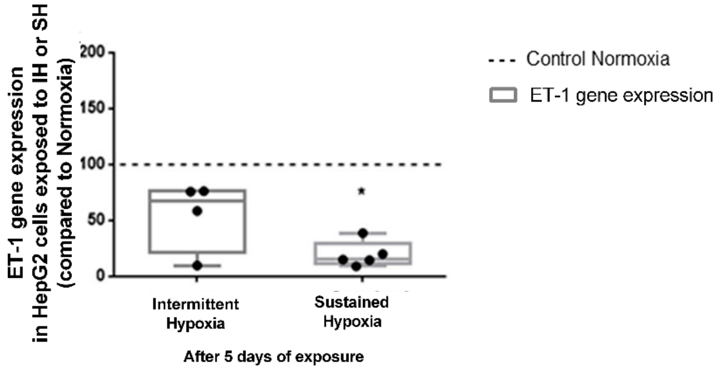

(a)

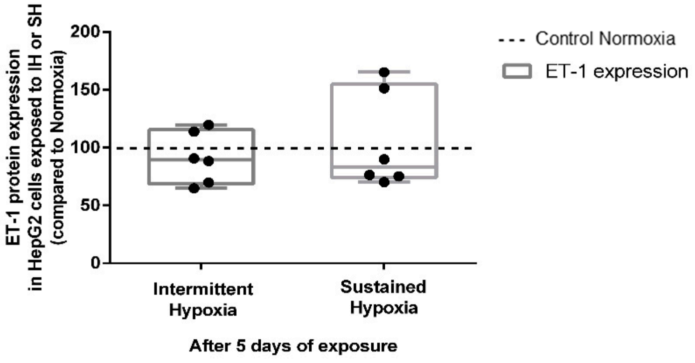

(b)

Supplementary Figure S1. In vitro effects of IH and SH on ET-1 gene expression and protein expression

(a). SH but not IH is associated with a significant decrease of ET-1 gene expression in HepG2 cells after 5 days of exposure ($p<0.05$). The levels of ET-1 gene expression was measured by RT-QPCR; n= 3 independent experiments/group. *$p<0.05$, Mann-Whitney test.

(b). Neither Intermittent Hypoxia nor Sustained Hypoxia modify ET-1 protein secretion by HepG2 cells after 5 days of exposure. The levels of Et-1 protein secreted was measured by Elisa on super natant; n= 3 independent experiments/group, Mann-Whitney test.

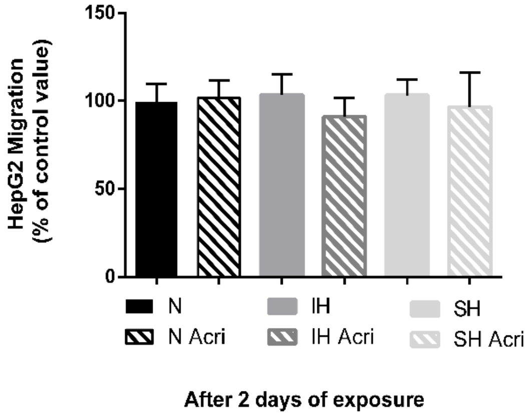

(a)

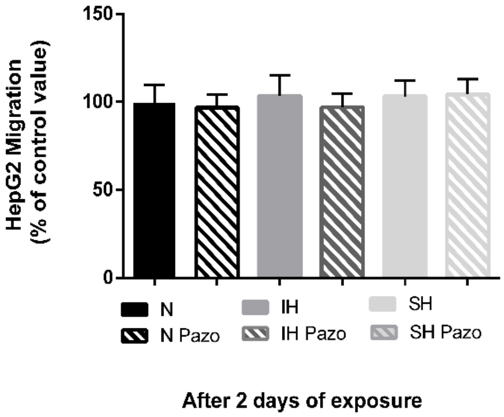

(b)

Supplementary Figure S2.

(a). Migration, expressed as a % of control values, of viable HepG2 cells after 2 days of Normoxia, Intermittent Hypoxia or Sustained Hypoxia and treated or not by acriflavine (acri); n=3 independent experiment/group (9 wells/group: 2 transwells/experiments). Mann-Whitney test. p NS, Mann-Whitney test.

(b). Migration, expressed as a % of control values, of viable HepG2 cells after 2 days of Normoxia, Intermittent Hypoxia or Sustained Hypoxia and treated or not by pazopanib (pazo); n=3 independent experiment/group (9 wells/group: 2 transwells/experiments). Mann-Whitney test. p NS, Mann-Whitney test.

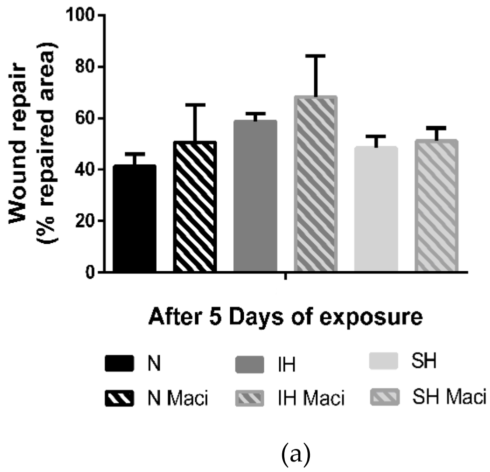

(a)

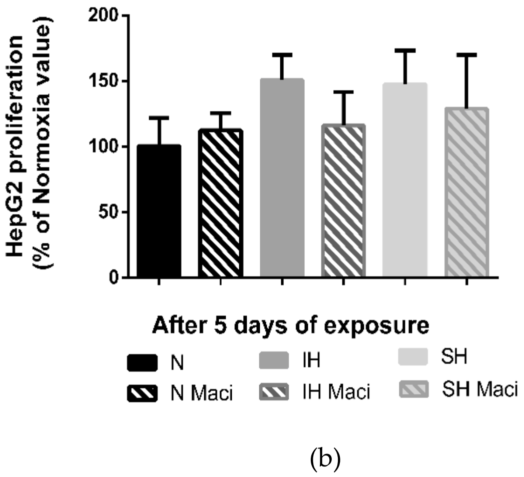

(b)

Supplementary Figure S3

(a). Wound healing repair, expressed as a % of repaired area, of HepG2 t least 3 wells/experiment after 5 days of Normoxia, Intermittent Hypoxia or Sustained Hypoxia and treated or not by macitentan (maci). p NS, Mann-Whitney test.

(b) Proliferation, expressed as a % of Normoxia, of viable HepG2 cells quantified by MTT staining after 5 days of Normoxia, Intermittent Hypoxia or Sustained Hypoxia and treated or not by macitentan (maci); n=3 independent experiment/group (6 wells/group: 2 transwells/experiments). p NS, Mann-Whitney test.
